# Opposite polarities of ENSO drive distinct patterns of coral bleaching potentials in the southeast Indian Ocean

**DOI:** 10.1038/s41598-017-02688-y

**Published:** 2017-05-26

**Authors:** Ningning Zhang, Ming Feng, Harry H. Hendon, Alistair J. Hobday, Jens Zinke

**Affiliations:** 1 CSIRO Oceans and Atmosphere, IOMRC, Crawley, Western Australia Australia; 2 School of Marine Sciences, Nanjing University of Information Science and Technology, Nanjing, China; 3 Western Australia Marine Science Institution, Perth, Western Australia Australia; 4 Bureau of Meteorology, Melbourne, Australia; 5 CSIRO Oceans and Atmosphere, Hobart, Tasmania Australia; 6 Institut für Geologische Wissenschaften, Freie Universität Berlin, Berlin, Germany; 7 Department of Environment and Agriculture, Curtin University, Bentley, Australia; 8 Australian Institute of Marine Science, Crawley, Australia; 9 School of Geography, Archaeology and Environmental Studies, University of Witwatersrand, Johannesburg, South Africa

## Abstract

Episodic anomalously warm sea surface temperature (SST) extremes, or marine heatwaves (MHWs), amplify ocean warming effects and may lead to severe impacts on marine ecosystems. MHW-induced coral bleaching events have been observed frequently in recent decades in the southeast Indian Ocean (SEIO), a region traditionally regarded to have resilience to global warming. In this study, we assess the contribution of El Niño-Southern Oscillation (ENSO) to MHWs across the mostly understudied reefs in the SEIO. We find that in extended summer months, the MHWs at tropical and subtropical reefs (divided at ~20°S) are driven by opposite ENSO polarities: MHWs are more likely to occur at the tropical reefs during eastern Pacific El Niño, driven by enhanced solar radiation and weaker Australian Monsoon, some likely alleviated by positive Indian Ocean Dipole events, and at the subtropical reefs during central Pacific La Niña, mainly caused by increased horizontal heat transport, and in some cases reinforced by local air-sea interactions. Madden-Julian Oscillations (MJO) also modulate the MHW occurrences. Projected future increases in ENSO and MJO intensity with greenhouse warming will enhance thermal stress across the SEIO. Implementing forecasting systems of MHWs can be used to anticipate future coral bleaching patterns and prepare management responses.

## Introduction

Acute disturbances to coral reefs and adjacent marine ecosystems are increasing in frequency and severity and altering coral cover and composition of marine benthic communities^1–4^. Episodic warm sea surface temperature (SST) extremes, termed marine heatwaves (MHWs), which are often associated with the most powerful climate phenomenon– El Niño-Southern Oscillation (ENSO)^5^, are among the most important environmental pressures that threaten the sustainability of coral reefs^6–8^. A large number of the world’s coral reefs were recently influenced by one of the most severe El Niño events on record that lasted from early-2014 to mid-2016, dubbed the Godzilla-El Niño^9^, causing severe coral bleaching worldwide, including in regions previously considered resilient to these effects, such as the southeast Indian Ocean (SEIO)^10^.

**Figure 1:**
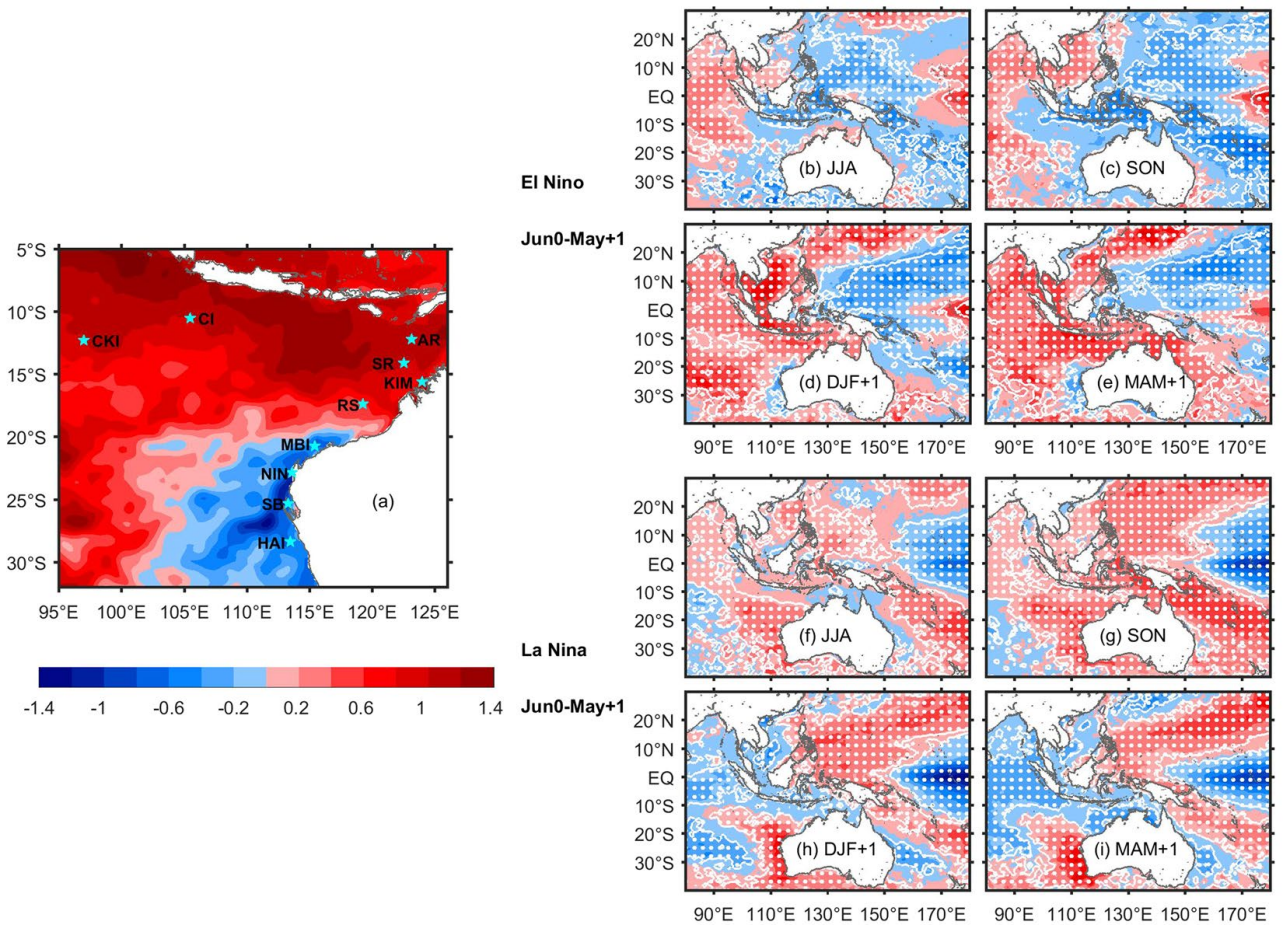
(**a**) Averaged SST anomalies in the SEIO during December 2015-April 2016. Stars denote locations of representative SEIO reefs. (**b**–**e**) Composited SST anomalies for the El Niño events during January 1982-April 2015 and (**f**–**i**) for the La Niña events in the same time period. The white contours and dots indicate anomalies exceeding the 95% significance level based on a two-tailed Student’s *t* test. JJA = June to August, SON = September to November, DJF = December to February of year 1, MAM = March to May of year 1. Figures are plotted using MATLAB R2015b (http://www.mathworks.com/). The maps in this figure are generated by MATLAB R2015b with M_Map (a mapping package, http://www.eos.ubc.ca/~rich/map.html).

The SEIO hosts a number of biodiverse coastal fringing and offshore oceanic atoll coral reefs^4, 11^ (Fig. 1a), and was considered to be under relatively low levels of duress from local human impacts and climate variability^12, 13^. In recent decades, however, the region has suffered from major coral bleaching events. During the 1997–98 El Niño event, the tropical Scott Reef was affected by extremely high thermal stresses of more than 13 degree heating weeks (DHW), causing 70–90% coral mortality^6, 14^. In contrast, the nearshore and most oceanic atoll reefs in the subtropical SEIO (south of 20°S) escaped bleaching during the 1997–98 event. During the 2010–11 La Niña, widespread coral bleaching was recorded across more than 12° of latitude south of the Montebello and Barrow Islands along the Western Australian (WA) coastline^15–17^. During the recent 2015–16 El Niño event, extremely high summer SSTs in the tropical SEIO (Fig. 1a) were reported to have caused 60–90% of the shallow coral community to bleach through April 2016^10^. These tropical-subtropical spatial contrasts suggest that bleaching response patterns in the SEIO are associated with opposite polarities of ENSO. In addition, SST anomalies associated with other climate change modes like the Indian Ocean Dipole (IOD), Madden-Julian Oscillations (MJO) and Interdecadal Pacific Oscillation (IPO) can also dampen or exacerbate the ENSO thermal stress levels in the SEIO^18–23^. Some reefs in the SEIO experienced bleaching during both ENSO polarities, such as the Cocos Keeling Island^24, 25^.

There have been comprehensive studies of the subtropical SEIO MHWs, in term of the roles of remote tropical Pacific drivers and local air-sea interaction^26–28^, however, the research on the MHWs in the tropical SEIO is less comprehensive, except for some cases studies^10, 29^. There is still a lack of knowledge on how the SST at different coral reefs responds to the opposite polarities and different flavours of ENSO such as eastern and central Pacific ENSO (EP-type and CP-type ENSO). Impacts from the interplay of the IOD and the MJO also need to be clarified. In this study, we investigate the SST response patterns to different polarities and flavours of ENSO in the extended austral summer season (December-April), when the SST are seasonally warmest and thus anomalies associated with ENSO can cause SST extremes. In particular, this study determines in which flavour and polarity of ENSO that MHWs preferentially occur and reveals the relative contributions of atmospheric and oceanic processes responsible for these SST extremes. This insight can be used to predict future coral bleaching patterns and prepare management responses^30^.

## Results

Partial correlation patterns show that SST variations in the tropical region of the SEIO are better correlated to ENSO variations that have maximum amplitude in the eastern Pacific, commonly referred to as EP-type ENSO or canonical ENSO^31, 32^. On the other hand, SST variations in the subtropical region of the SEIO are better correlated to ENSO variations that have maximum amplitude in the central Pacific, commonly referred to as CP-type ENSO, or ENSO Modoki^31, 32^ (Supplementary Fig. S1). Hence, we define El Niño and La Niña years based on EP-type El Niño and CP-type La Niña, respectively. Meanwhile we should note that these two types of ENSO are not completely independent of each other.

In the developing year of an El Niño, SST anomalies in the Indian Ocean are indicative of positive IOD events, which often develop during austral winter/spring in conjunction with El Niño^33^ (Supplementary Table S1, 2015 is defined as a neutral IOD year as the IOD eastern pole is used to define the IOD events), and cold SST anomalies appear off northern Australia (Fig. 1b,c). As El Niño matures in austral summer, SST anomalies off northern Australia switch to positive, and then strengthen and expand to a large tropical region during the autumn (Fig. 1d,e). Cold SST anomalies persist southward of 20°S along the WA coast. After removing the El Niño effects, a positive IOD event causes significant cold SST anomalies of more than 0.4 °C/°C in tropical SEIO in preceding austral winter and at some tropical places last in the preceding spring (Fig. S2). Although an IOD event may not have direct effects on the summer SST anomalies in the tropical SEIO (Fig. S2), it may precondition the coupled air-sea processes that drive the SST anomalies and thus may alleviate the warming extent in summer when co-occurs with an El Niño event (Supplementary Fig. S2).

During La Niña, positive SST anomalies first establish off the northwest coast of Australia during winter and spring, and then shift southward down the coast during austral summer, with notably high SST anomalies southward of 20°S at the mature phase of La Niña (Fig. 1f–i). Lower than normal SSTs occupy the northern coast in summer.

**Figure 2:**
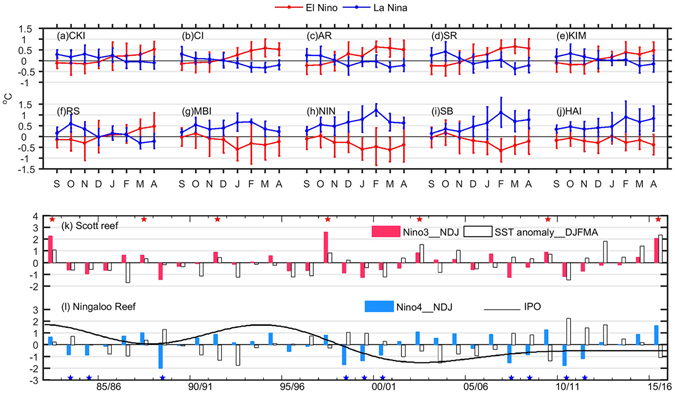
(**a**–**j**) Composite SST anomalies during El Niño and La Niña events at ten selected reefs. The error bars denote 95% confidence intervals. (**k**) Time series of standardised SST anomaly averaged in extended summer months (December-April) at the Scott reef and standardised November-January averaged Niño 3 index; (**l**) time series of standardised SST anomaly averaged in extended summer months (December-April) at the Ningaloo reef, standardised Niño4 index and IPO index. The red (blue) stars in (**k**,**l**) denote the developing years of El Niño (La Niña). Figures are plotted using MATLAB R2015b (http://www.mathworks.com/).

Among 10 representative tropical/subtropical reef sites in the SEIO (Fig. 1a), those located in the tropics (CKI, CI, AR, SR, KIM and RS) tend to have positive SST anomalies in the extended austral summer months during El Niño; whereas those in the subtropics (MBI, NIN, SB and HAI) tend to have positive SST anomalies in austral summer months during La Niña (Fig. 2a–j). The magnitude of anomalous warming varies with the intensity of each event and is influenced by intraseasonal variations (Supplementary Fig. S3). Positive SST anomalies also occur at the CKI in early summer during La Niña, which makes it susceptible to coral bleaching during both polarities of ENSO. An asymmetry exists between the magnitudes of positive SST anomalies caused by opposite polarities of ENSO: positive SST anomalies at the tropical reefs during El Niño are usually weaker than those at the subtropical reefs during La Niña.

Summer SST anomalies at the Scott Reef are positively correlated with the November-January averaged Niño3 index (correlation coefficient *r* = 0.5), and the SST anomalies show a warming tendency, with a maximum reaching more than 1 °C in the summer of 2015/16 (Figs 2 and S4a). Conversely, the summer SST anomalies at the Ningaloo Reef are negatively correlated with the Niño4 index (correlation coefficient *r* = −0.68), and all the La Niña years are associated with positive SST anomalies, with a maximum reaching 2 °C in 2010–11 (Figs 2l and S4b). The 2010–11 Ningaloo Niño MHW was associated with an extraordinarily strong La Niña in the Pacific Ocean and amplified by local air-sea coupling^16, 26^, and was followed by another two warmer than normal summers (Fig. 2), causing severe coral bleaching events in the region^15–17^. The SST anomaly series at the Ningaloo Reef also have significant decadal variation, with more warming events after the 1997–98 El Niño (Fig. 2), in conjunction with a phase shift of the IPO towards a cold phase that acts to promote warm conditions off the WA coast^21, 22, 27, 34^.

Composite patterns may smooth out intense short-term events, such that the MHW event at the Scott Reef in 1997–98 was not well captured by the seasonally averaged SST anomalies (Figs 2 and S4a). To characterise sub-seasonal features, we quantify episodic MHW events using daily data, defined as periods when surface temperatures are warmer than the 90^th^ percentile and last for five or more days based on a 30-year historical baseline period^35^.

**Figure 3:**
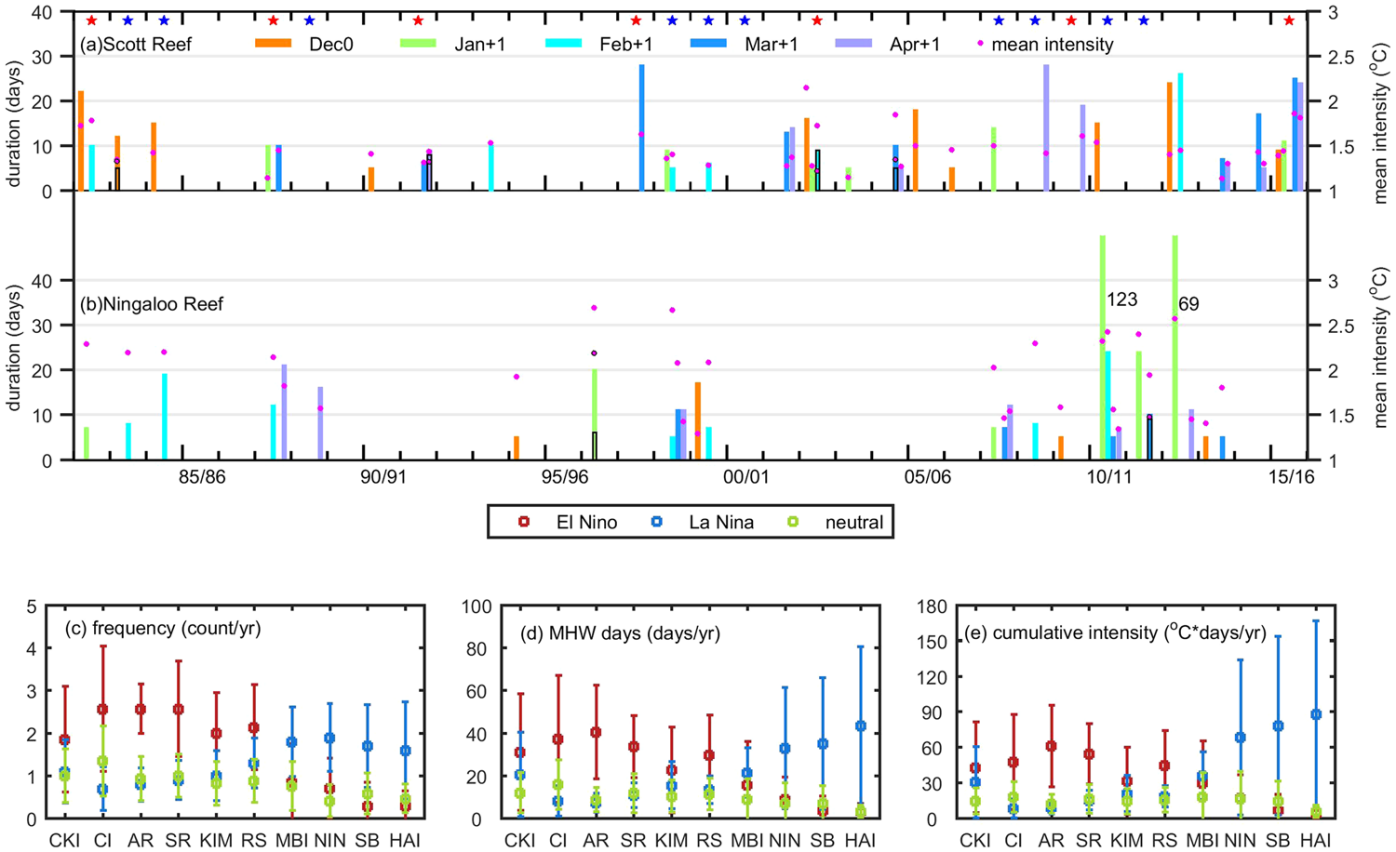
(**a**,**b**) Duration (bars) and mean intensity (dots) of MHW events peaking in extended summer months (December-April) at the Scott and Ningaloo reefs, respectively. The time axis corresponds to the peak month of each MHW event. The red (blue) stars denote the developing years of El Niño (La Niña). (**c**–**g**) Composited characteristics of MHW events at the 10 reefs in the extended summer months (December-April) of El Niño and La Niña events. The error bars denote 95% confidence intervals. Figures are plotted using MATLAB R2015b (http://www.mathworks.com/).

At the Scott Reef, the longest MHW event (28 days) occurred during March-April 1998, with a mean intensity of 1.63 °C (Fig. 3a) and a cumulative intensity of 46 °C·days (equal to 6.6 DHWs; Supplementary Table S1). The cumulative intensity is similar to DHW, which is frequently used to monitor coral bleaching, and a value of 4 DHWs could cause bleaching to occur^36^. In the recent 2015–16 El Niño, frequent MHW events with both long durations and strong intensities occurred across all the tropical sites, in sharp contrast to almost none at the subtropical sites (Supplementary Table S1; Figs 3 and S5). On the other hand, at the Ningaloo Reef, the MHW events were mostly associated with La Niña events (Fig. 3b), such as during the 1998–99, 2007–08, 2010–12 La Niña events. The longest MHW at the Ningaloo Reef developed from September 2010 through January 2011 and then was followed by another MHW event lasting for almost a month in February 2011, amounting to a total cumulative intensity of 361 °C·days in the extended summer months (Supplementary Table S1). Overall, there were also increasing frequencies of short-term MHWs during 1982–2016 (Fig. 3a,b).

The MHWs at opposite ENSO polarities show distinct contrasts between the tropical and subtropical reefs (Fig. 3c–e). For the tropical reefs, the frequency, MHW days, and cumulative intensity are all higher during El Niño; while for the subtropical reef sites, these properties are significantly higher during La Niña. However, the duration and mean intensity of individual events do not show such contrasts for opposite polarities of ENSO (Supplementary Fig. S6).

**Figure 4:**
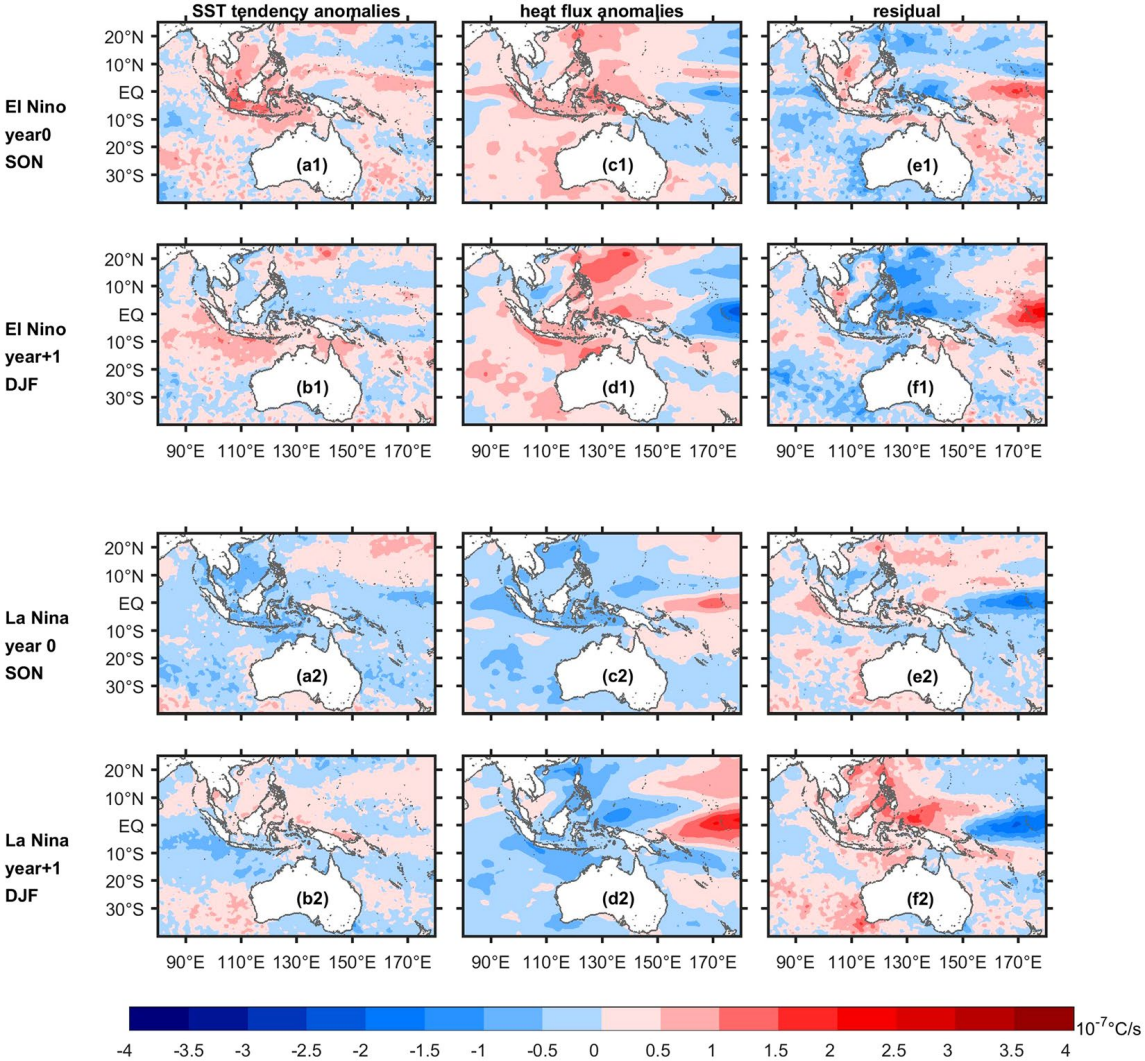
(**a1**–**f1**) Mixed layer heat budget terms during spring (SON) and summer (DJF) for El Niño events, and (**a2**–**f2**) for La Niña events. SON = September to November, DJF = December to February. Figures are plotted using MATLAB R2015b (http://www.mathworks.com/). The maps in this figure are generated by MATLAB R2015b with M_Map (a mapping package, http://www.eos.ubc.ca/~rich/map.html).

Anomalous warm conditions at the tropical reefs are mainly caused by local atmospheric forcing in responses to the EP-type El Niño teleconnection, which increases surface heat flux into the ocean^33^ (Fig. 4a1–f1), whereas warm conditions at the subtropical reefs were mainly attributed to oceanic processes such as increased horizontal heat advection^26, 27, 37^ (Fig. 4a2–f2).

**Figure 5:**
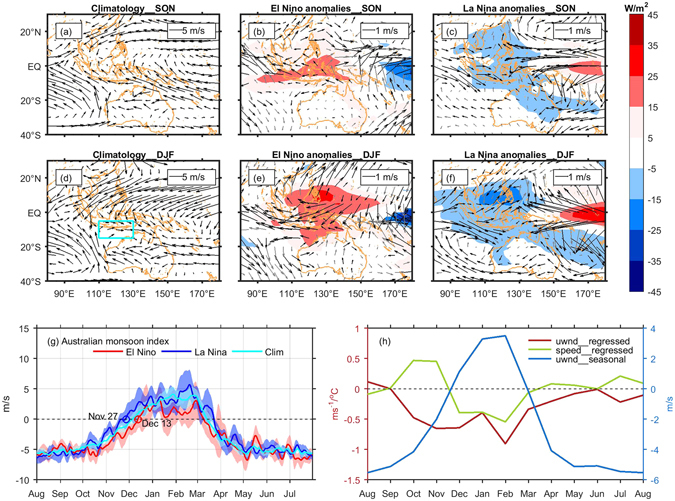
(**a**–**f**) Climatological mean, anomalous 925 hPa wind (arrows) and OLR anomalies (shaded) during spring and summer of opposite ENSO polarities. Anomalies exceeding the 90% significant level based on a two-tailed Student’s *t* test are displayed (black arrows for significant wind anomalies and grey for insignificant ones). The cyan box in (**d**) indicates the region where the Australian monsoon index is defined. (**g**) The composites of the Australian monsoon wind index. The red and blue circles are onset dates of Australian summer monsoon during El Niño and La Niña respectively. The shadows denote corresponding 95% confidence intervals. (**h**) Lagged regression of 850 hPa zonal component of wind and wind speed averaged in the box indicated in (**g**) onto Niño3 index in January and seasonal cycle of 850 hPa zonal component of wind averaged in the box. Figures are plotted using MATLAB R2015b (http://www.mathworks.com/). The maps in this figure are generated by MATLAB R2015b with M_Map (a mapping package, http://www.eos.ubc.ca/~rich/map.html).

The increase of surface heat flux in the tropical SEIO during El Niño is mainly due to shortwave radiation and latent heat anomalies (Supplementary Fig. S7), stemming from the variation of the Walker circulation^33, 38^. During El Niño, the eastward displacement of the ascending branch of the Walker circulation in the Pacific results in less convective cloud coverage over the Maritime Continent and eastern tropical Indian Ocean, as indicated by positive outgoing longwave radiation anomalies (OLR, Fig. 5b,e), thereby increasing the incident shortwave radiation by more than 15 Wm^−2^ in the tropical SEIO (Supplementary Fig. S7)^39^. This reduced convection acts to enhance the trade winds across the tropical SEIO. And onset of the Australian summer monsoon is delayed and weakened^40^ (Fig. 5g,h). In the tropical region off the south coast of Java-Lesser Sunda Islands, the anomalous easterlies during El Niño years thus acts to increase latent heat loss and promote cooling in the pre-monsoon season (spring), but act to promote warming during summer once the monsoon onsets^38–40^ (Supplementary Fig. S7). Modification of the Walker circulation caused by EP-type ENSO can extend to tropical region of the SEIO, while the variation centres of convection for CP-type ENSO are mainly limited within the tropical Pacific region, so SST variability in the SEIO is more closely correlated to the EP-type El Niño^31, 32^.

The subtropical MHWs, the “Ningaloo Niño”, is closely tied to the variability of the Leeuwin Current (LC). The LC flows against the prevailing southerly wind along the west coast of Australia^26–28, 41^. The LC is affected by ENSO from the western Pacific via coastal Kelvin waves: transport decreases (increases) in El Niño (La Niña) years^26, 27, 41^ bringing less (more) heat southward, thus decreasing (increasing) the local SST. The LC variations appear to be more sensitive to the SST gradient between the Niño4 region and western tropical Pacific^7, 42^, thus SST variations in the subtropical region of the SEIO are more closely related to the CP-type ENSO. In addition to the response to remote forcing from the Pacific, amplification of the LC transport and coastal downwelling due to the collapse of the southerly winds also enhances warming off the WA in some years^26–28, 43^. During the locally-amplified Ningaloo Niño, such as in years 2010–11 and 2011–12, cyclonic wind anomalies developed to the northwest of Australia in September (Supplementary Fig. S8), and extended south-eastward in summer, reducing wind speed and evaporation related heat loss (Supplementary Fig. S8), and inducing coastal downwelling anomalies, thus reinforcing the positive SST anomalies^27, 28, 37, 43^. The MHWs at the subtropical reefs in these two years were unusually strong and long-lasting (Fig. 3, Supplementary Table S1, Supplementary Fig. S5).

**Figure 6:**
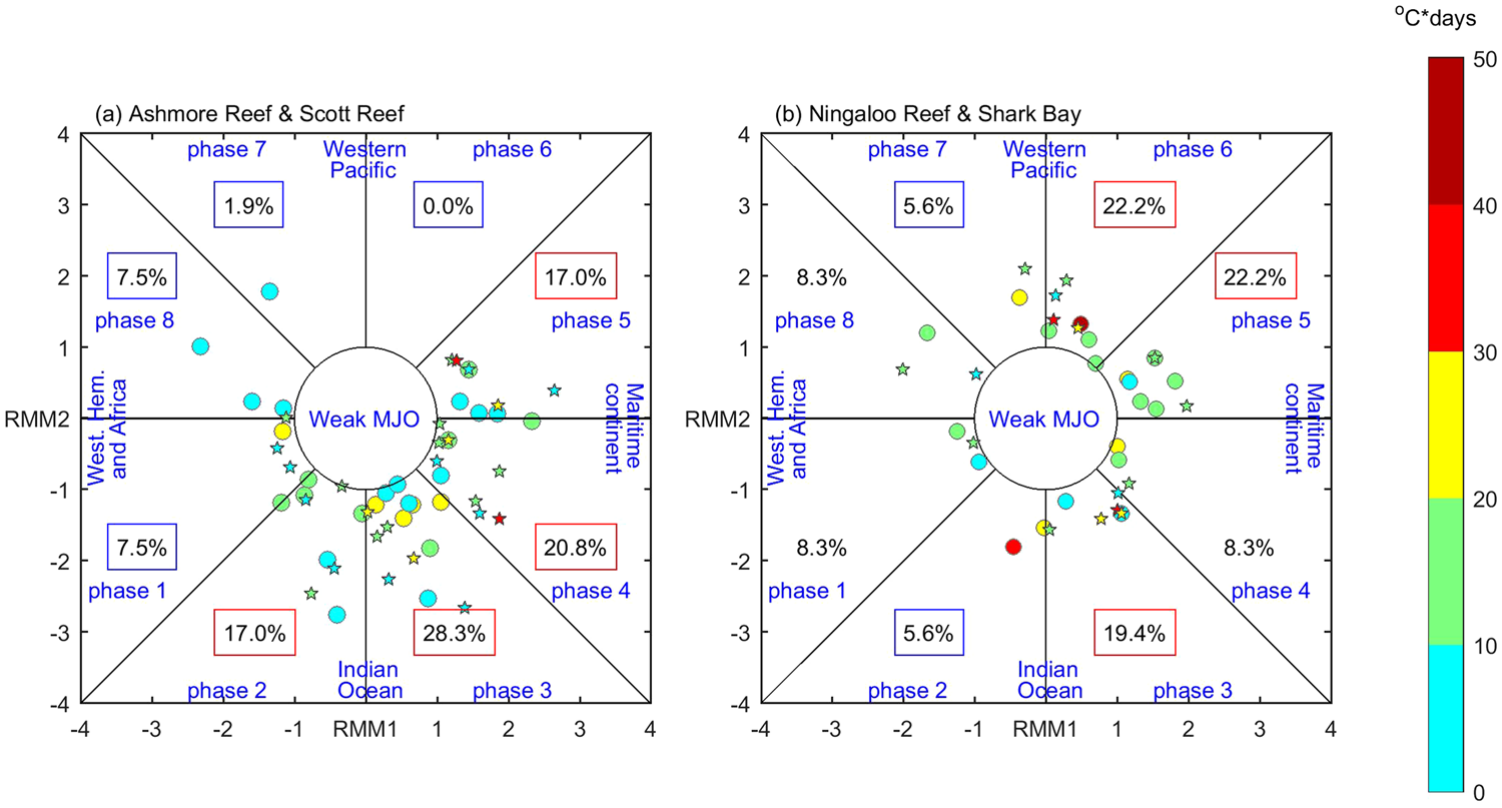
Distribution of the MHW peaks in extended summer months as a function of MJO phases at (**a**) the tropical Ashmore (circles) and Scott Reefs (stars) and (**b**) subtropical Ningaloo Reef (circles) and Shark Bay (stars). The percentages stand for the proportions of MHW peaks in that phase of strong MJO and are boxed in red (blue) where they are significantly greater (less) than the average occurrence (8.3%). Only MHWs with duration of less than 20 days are shown. Figures are plotted using MATLAB R2015b (http://www.mathworks.com/).

MJOs also affect SST variations in the SEIO by modulating the local atmospheric conditions^20, 44^. A clear MJO-footprint can be seen from the distributions of MHW peaks in different MJO phases (Fig. 6). At the tropical reefs such as the Ashmore and Scott Reefs, there tend to be more MHWs peaking at MJO phases 2–5 and less in the other phases. Phases 2–5 coincide with suppressed convection, anomalous heat flux into the ocean and Ekman-induced downwelling off the northwest Australian shelf, thus increasing the local SST^20^. The other phases of the MJO (active convection) are associated with surface heat flux cooling and Ekman-induced upwelling^20^, thus were not favourable for MHWs. For the subtropical reefs, such as the Ningaloo Reef and Shark Bay, there tend to be more MHWs peaking in MJO phase 3–6, although the modulation is weaker than for the northern reefs because the MJO has its biggest impacts near the equator. The increased MHWs in phases 3–6 stems from the accumulation of warm water off the northwest Australian shelf, which triggers southward propagating Kelvin waves that increase the LC heat transport^20^.

## Discussion

In the tropical region of the SEIO, strong MHWs often tend to occur in extended summer months during EP-type El Niño, modulated by preceding cooling in winter and spring when positive IOD events co-occur; while in subtropical region, long-lasting and strong MHWs tend to occur in extended summer months during CP-type La Niña. In both regions, there tend to be more MHWs in the suppressed convection phases of MJO. In response to increasing greenhouse gases, extreme El Niño and La Niña events are projected to increase^45–47^. MJOs are also projected to be more active under global warming^48, 49^. Thus, there will be increased likelihood of MHW disturbances in the SEIO, posing greater threat to the ecosystem. The IOD events are normally phase locked with austral winter and spring, but there are also intrinsic IOD events that mature earlier in austral winter^50, 51^. Further research is needed to distinguish the effects of different types of IOD events on MHWs in the SEIO.

Our work shows that under the influence of different flavours of ENSO, and modified by other climate modes like IOD, MJO, IPO and local air-sea interactions, the coral bleaching potentials vary across the SEIO, and may occur more frequently in a warming world^52, 53^. Performances of both dynamical and statistical prediction models suggest that ENSO predictive skills have been improving, implying predictability of ENSO-induced variabilities such as MHWs^37^. For instance, the Predictive Ocean Atmosphere Model for Australia (POAMA) well predicted the patterns of SST and sea surface height (SSH) anomalies in March 2016^54^), providing the possibility to predict increased risk of MHWs at least one season ahead. Such a lead time can allow time to plan for deployment of monitoring activities^30^ and help as an early warning system for potential bleaching^55^. Forecasting MHWs can also reduce coral recovery times by extending the time available to implement ways to mitigate local scale stress from human-related activities at severely impacted sites, such as restrictions of fishing or tourism activities. In future, seasonal forecasts can also be used to support preparation of active interventions that require long lead times, such as predator control programs (e.g. removal of crown of thorns) or out-planting of new coral propagules, which may enhance reef resilience in the face of a changing climate.

## Data

The NOAA 1/4° daily optimally interpolated SST (OISST) data for January 1^st^ 1982-May 31^st^ 2016 used in this analysis are acquired from NOAA’s National Centers for Environmental Information (NCEI) at https://www.ncdc.noaa.gov/oisst/data-access. The Niño 3 and Niño 4 indexes are obtained from the Ocean Observations Panel for Climate (OOPC). The IPO index is obtained from the NOAA’s Earth System Research Laboratory (http://www.esrl.noaa.gov/psd/data/timeseries/IPOTPI/)^56^. The Real-time Multivariate MJO series (RMM) are obtained from the International Research Institute (IRI) for Climate and Society Data Library (http://iridl.ldeo.columbia.edu/SOURCES/.BoM/.MJO/.RMM/). The same 8 phases defined by Wheeler and Hendon (2004) are used^57^. The 925 hPa and 850 hPa winds are obtained from NCEP Reanalysis 2 data provided by the NOAA/OAR/ESRL PSD, Boulder, Colorado, USA, from their Web site at http://www.esrl.noaa.gov/psd/, for the period January 1982- April 2016^58^. The NOAA interpolated outgoing longwave radiation (OLR) are used as proxy for cloud cover. Heat fluxes from the ERA-Interim reanalysis during January 1982- March 2016 are used to conduct temperature budget analyses in the upper 50 m^59^.

## Methods

10 representative tropical/subtropical reef sites in the SEIO are selected for this study: the Cocos Keeling Island (CKI), Christmas Island (CI), Ashmore Reef (AR), Scott Reef (SR), Kimberley (KIM), Rowley Shoals (RS), Montebello and Barrow Islands (MBI), Ningaloo Reef (NIN), Shark Bay (SB) and Houtman Abrolhos Island (HAI) (Fig. 1a).

### Definition of El Niño and La Niña years

The Niño 3 and Niño 4 indexes are used to identify the EP-type El Niño and CP-type La Niña events, respectively (Supplementary Table S1), since the summer SST variations in the tropical and subtropical SEIO were sensitive to the SST anomalies in the eastern and central equatorial Pacific respectively^7, 27, 53^ (Supplementary Fig. S1).

The El Niño years are defined using the Niño3 index, following NOAA tradition, as when the running 3-month mean SST anomaly in the Niño3 region are above 0.5 °C for at least 5 consecutive months; the La Niña years are identified when the running 3-month mean SST anomaly in the Niño4 region are below −0.5 °C for at least 5 consecutive months.

### Definition of a marine heatwave event

A marine heatwave event is defined as a discrete prolonged anomalously warm water event in a particular location^35^. The relative baseline climatology is defined using all data within an 11-day window centered on the time of year with a period of 30 years and then a high percentile threshold (90% in our paper) is used to obtain the threshold value. A percentile threshold rather than an absolute value above the climatology is used since the magnitude of SST variability varies by different regions. “Prolonged” means a MHW must last more than 5 days and “discrete” means two events with a gap of 2 days or less will be considered as a continuous event. The MHW days in this paper are the number of days that anomalous warming happens in the austral summer period (December-April), and cumulative intensity is the integral of intensity over the duration of the event.

### Mixed-layer temperature budget

The temperature budget in the upper layer can be estimated from the following equation^60^,

1 $$\frac{\partial T}{\partial t}=\frac{{Q}_{0}-{q}_{d}}{\rho {C}_{p}{h}_{u}}+Residual$$

where *T* is the SST, *ρ* is the reference density (1026 kg m^−3^), *C*_*p*_ is the specific heat capacity of seawater (4000 J kg^−1^ K^−1^), and *h*_*u*_ is the upper layer depth (designated to 50 m). The terms in equation (1) are referred to as the SST tendency, surface heat flux, and residual, respectively. The residual term includes the effects of horizontal advection and entrainment, mostly dominated by advection in our study area.

*Q*_0_ is the net surface heat flux, including the shortwave radiation, longwave radiation, latent and sensible heat flux:

$${Q}_{0}=SW+LW+LH+SH,$$

*q*_*d*_ is the downward radiative flux across the upper layer, which is estimated as

$${q}_{d}=SW[R{e}^{(-\frac{{h}_{u}}{{r}_{1}})}+(1-R){e}^{(-\frac{{h}_{u}}{{r}_{2}})}],$$

where SW is shortwave radiative heat flux; *R*, *r*_*1*_ and *r*_*2*_ are coefficients that depend on water turbidity, which are selected to be 0.58, 0.35 and 23 here^60^.

### Australian monsoon index and coastal wind index

The Australian monsoon is described by the Australian monsoon index (AUSMI), which is defined as 850 hPa zonal wind averaged over the area (5°S–15°S, 110°E–130°E)^28^. The onset date of the Australian summer monsoon is defined followed the criteria of Kajikawa *et al*.^38^. The coastal wind index (CWI) is defined as the 925 hPa meridional wind anomalies averaged over the region 108°E to the coast, 28° to 22°S in austral summer (December-February)^28^. It defines whether or not there are significant northerly wind anomalies off the WA coast^28^. According to the CWI, the ten La Niña events since 1982 can be classified into two types– locally amplified and non-locally amplified modes^28, 37, 43^. For the non-locally amplified mode, there were no significant alongshore wind anomalies thus no contribution to the warming off the WA (Supplementary Fig. S8). The local alongshore wind anomalies were indicated to be largely affected by the Australian summer monsoon and Southern Annular Mode (SAM), and anomalous northerly alongshore winds in the locally amplified mode were often accompanied by weaker Australian summer monsoon compared to the non-locally amplified mode and positive SAM^28, 43^.

## Electronic supplementary material

Supplementary figures
