# Supplementary material for: Opposite polarities of ENSO drive distinct patterns of coral bleaching potentials in the southeast Indian Ocean
Source: Sci Rep. 2017 May 26;7:2443. doi: 10.1038/s41598-017-02688-y (PMC5446420; doi:10.1038/s41598-017-02688-y)
Supplement: Supplementary file 1 — Supplementary figures [file 41598_2017_2688_MOESM1_ESM.docx]

**Opposite polarities of ENSO drive distinct patterns of coral bleaching potentials in the southeast Indian Ocean**

**Ningning Zhang^1,2^, Ming Feng^1,3*^, Harry H. Hendon^4^, Alistair J. Hobday^5^, Jens Zinke^6,7,8,9^**

^1^CSIRO Oceans and Atmosphere, IOMRC, Crawley, Western Australia, Australia

^2^School of Marine Sciences, Nanjing University of Information Science and Technology, Nanjing, Jiangsu, China

^3^Western Australia Marine Science Institution, Perth, Western Australia, Australia

^4^Bureau of Meteorology, Melbourne, Australia

^5^CSIRO Oceans and Atmosphere, Hobart, Tasmania, Australia

^6^Institut für Geologische Wissenschaften, Freie Universität Berlin, Berlin, Germany

^7^Department of Environment and Agriculture, Curtin University, Australia

^8^Australian Institute of Marine Science

^9^School of Geography, Archaeology and Environmental Studies, University of Witwatersrand, Johannesburg, South Africa

^*^ *Corresponding author address:* Ming Feng, CSIRO Oceans and Atmosphere Research, IOMRC, Crawley, Western Australia, Australia. E-mail: ming.feng@ csiro.au

**Supplementary Table. S1** Cumulative intensities of each site in each year. The ten numbers in each cell correspond to CKI, CI, AR, SR, KIM, RS, MBI, NIN, SB and HBI respectively (see the right bottom cells). Unit: ºC⋅days.

|  | El Niño | | | | | | Neutral | | | | | | La Niña | | | | | |
| --- | --- | --- | --- | --- | --- | --- | --- | --- | --- | --- | --- | --- | --- | --- | --- | --- | --- | --- |
| pIOD | 1982/1983 | 103 | 65 | 53 | 56 | 29 | 1994/1995 | 0 | 5 | 9 | 0 | 0 | 2007/2008 | 12 | 12 | 9 | 21 | 28 |
|  |  | 82 | 48 | 47 | 48 | 15 |  | 9 | 12 | 10 | 17 | 0 |  | 43 | 46 | 43 | 33 | 68 |
|  | 1991/1992 | 0 | 16 | 23 | 31 | 0 | 2006/2007 | 18 | 26 | 0 | 7 | 12 | 2008/2009 | 0 | 0 | 28 | 40 | 40 |
|  |  | 28 | 0 | 0 | 0 | 0 |  | 17 | 0 | 0 | 0 | 0 |  | 26 | 35 | 18 | 0 | 0 |
|  | 1997/1998 | 20 | 55 | 52 | 46 | 15 | 2012/2013 | 42 | 34 | 63 | 104 | 46 | 2011/2012 | 119 | 37 | 0 | 0 | 0 |
|  |  | 7 | 0 | 0 | 0 | 0 |  | 75 | 179 | 194 | 150 | 41 |  | 0 | 32 | 90 | 166 | 137 |
|  |  |  |  |  |  |  |  |  |  |  |  |  |  |  |  |  |  |  |
|  |  |  |  |  |  |  |  |  |  |  |  |  |  |  |  |  |  |  |
| Neutral | 1987/  1988 | 8 | 0 | 16 | 26 | 18 | 1985/1986 | 0 | 0 | 0 | 0 | 0 | 1983/1984 | 11 | 0 | 0 | 23 | 22 |
|  |  | 62 | 131 | 64 | 0 | 12 |  | 0 | 7 | 0 | 0 | 0 |  | 25 | 0 | 18 | 18 | 0 |
|  | 2002/2003 | 36 | 0 | 75 | 67 | 41 | 1986/1987 | 0 | 6 | 15 | 0 | 0 | 1984/1985 | 0 | 0 | 13 | 21 | 11 |
|  |  | 0 | 0 | 0 | 0 | 0 |  | 0 | 0 | 0 | 0 | 0 |  | 27 | 35 | 42 | 33 | 15 |
|  | 2009/2010 | 5 | 43 | 55 | 31 | 6 | 1989/1990 | 0 | 0 | 16 | 0 | 14 | 1988/1989 | 0 | 6 | 0 | 0 | 6 |
|  |  | 29 | 0 | 8 | 0 | 0 |  | 13 | 0 | 0 | 22 | 12 |  | 33 | 24 | 25 | 30 | 0 |
|  | 2015/2016 | 87 | 155 | 156 | 125 | 113 | 1990/1991 | 0 | 0 | 0 | 7 | 7 | 1999/2000 | 8 | 6 | 6 | 8 | 0 |
|  |  | 106 | 30 | 0 | 0 | 0 |  | 12 | 0 | 0 | 0 | 0 |  | 7 | 17 | 37 | 13 | 55 |
|  |  |  |  |  |  |  | 1993/1994 | 0 | 0 | 0 | 15 | 0 | 2000/2001 | 0 | 0 | 7 | 0 | 0 |
|  |  |  |  |  |  |  |  | 0 | 0 | 0 | 0 | 0 |  | 6 | 0 | 0 | 0 | 0 |
|  |  |  |  |  |  |  | 1995/1996 | 0 | 7 | 0 | 0 | 0 |  |  |  |  |  |  |
|  |  |  |  |  |  |  |  | 0 | 0 | 0 | 0 | 0 |  |  |  |  |  |  |
|  |  |  |  |  |  |  | 2003/2004 | 7 | 0 | 5 | 6 | 0 |  |  |  |  |  |  |
|  |  |  |  |  |  |  |  | 0 | 0 | 0 | 0 | 0 |  |  |  |  |  |  |
|  |  |  |  |  |  |  | 2004/2005 | 7 | 84 | 30 | 32 | 80 |  |  |  |  |  |  |
|  |  |  |  |  |  |  |  | 54 | 7 | 0 | 0 | 0 |  |  |  |  |  |  |
|  |  |  |  |  |  |  | 2014/2015 | 67 | 54 | 12 | 31 | 30 |  |  |  |  |  |  |
|  |  |  |  |  |  |  |  | 36 | 0 | 0 | 8 | 12 |  |  |  |  |  |  |
| nIOD |  | | | | | | 1992/1993 | 0 | 0 | 0 | 0 | 0 | 1998/1999 | 35 | 0 | 16 | 19 | 79 |
|  |  |  |  |  |  |  |  | 0 | 0 | 0 | 0 | 0 |  | 0 | 64 | 52 | 98 | 229 |
|  |  |  |  |  |  |  | 1996/1997 | 7 | 11 | 0 | 0 | 0 | 2010/2011 | 122 | 26 | 13 | 23 | 23 |
|  |  |  |  |  |  |  |  | 10 | 57 | 67 | 20 | 18 |  | 19 | 111 | 361 | 392 | 379 |
|  |  |  |  |  |  |  | 2001/2002 | 26 | 0 | 9 | 36 | 8 |  |  |  |  |  |  |
|  |  |  |  |  |  |  |  | 7 | 0 | 0 | 0 | 0 |  |  |  |  |  |  |
|  |  |  |  |  |  |  | 2005/2006 | 7 | 8 | 32 | 27 | 45 |  |  |  |  |  |  |
|  |  |  |  |  |  |  |  | 0 | 8 | 0 | 10 | 10 |  |  |  |  |  |  |
|  |  |  |  |  |  |  | 2013/2014 | 71 | 74 | 20 | 16 | 7 | year | CKI | CI | AR | SR | KIM |
|  |  |  |  |  |  |  |  | 33 | 36 | 16 | 21 | 11 |  | RS | MBI | NIN | SB | HBI |


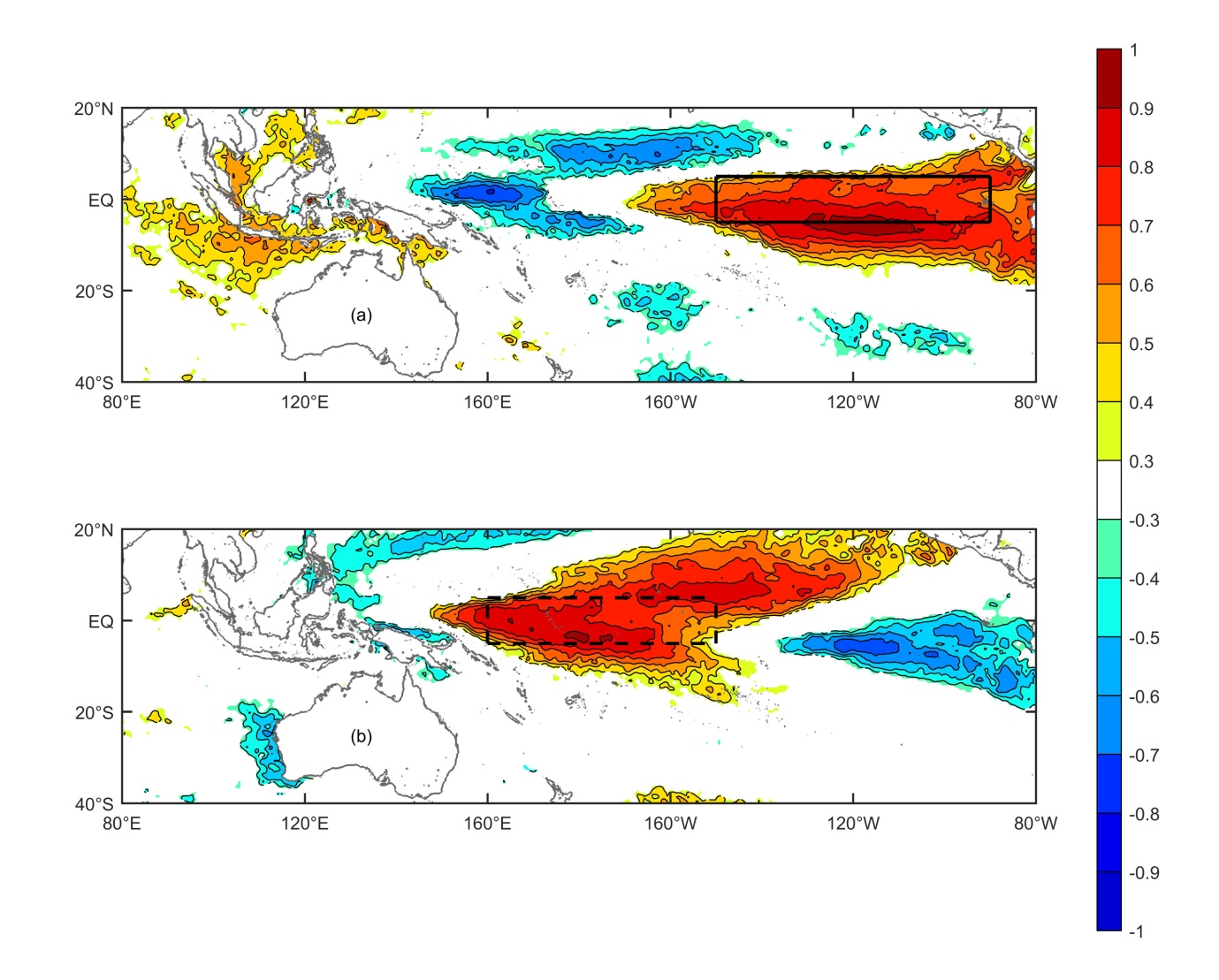


**Supplementary Fig. S1.** **(a)** Partial correlation coefficients between February-April averaged SST anomalies and December-February averaged Niño3 index (after the covariances with Niño4 and Dipole Mode Index at 0 month lead are removed, and solid-line box indicates Niño3 region ) and **(b)** February-April averaged SST anomalies and December-February averaged Niño4 index (after the covariances with Niño3 and Dipole Mode Index at 0 month lead are removed, and the dashed-line box indicates Niño4 region). Figures are plotted using MATLAB R2015b (<http://www.mathworks.com/>). The maps in this figure are generated by MATLAB R2015b with M_Map (a mapping package, <http://www.eos.ubc.ca/~rich/map.html>).


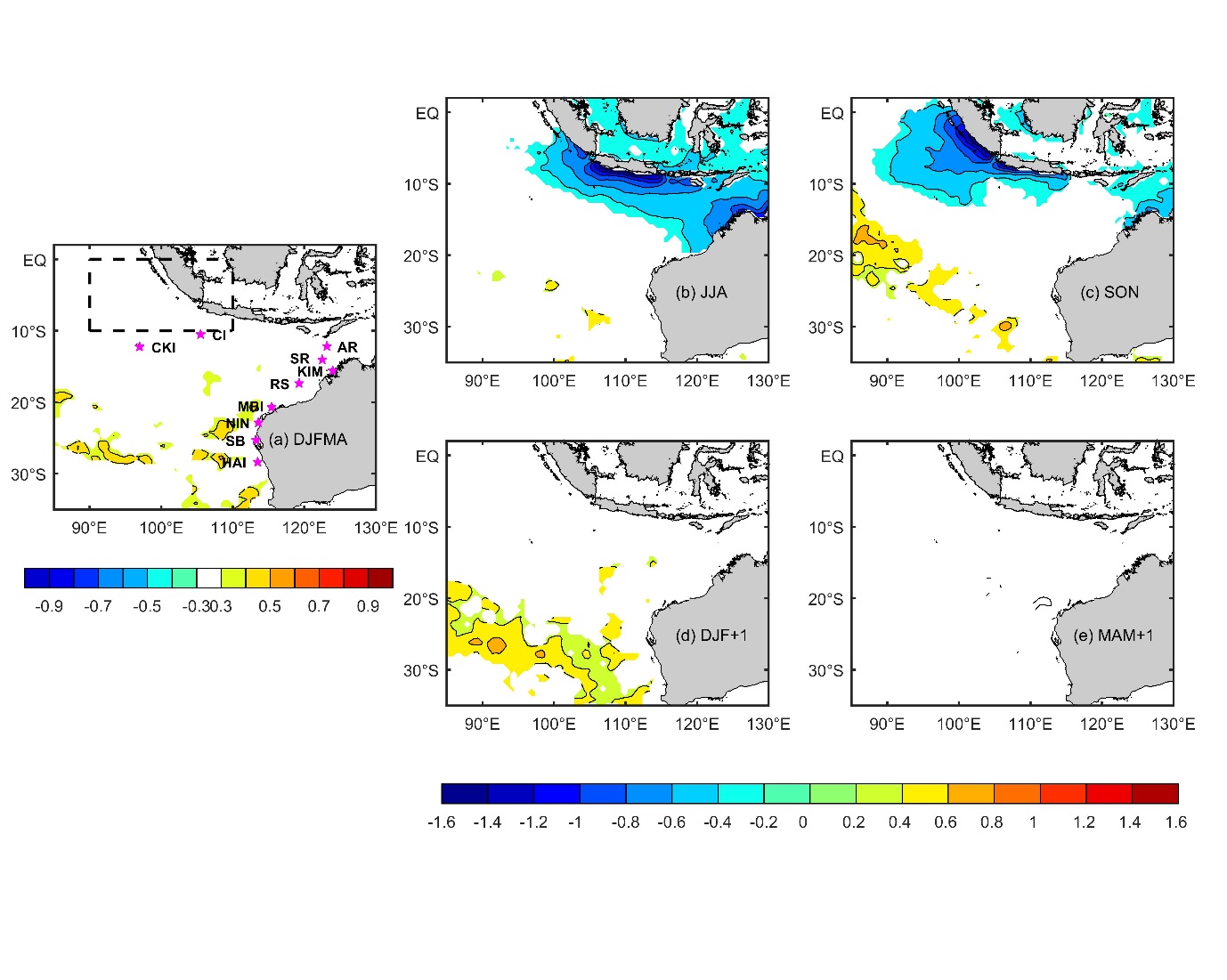


**Supplementary** **Fig. S2. (a)** Partial correlation between December-April averaged SST anomalies and September-November averaged Dipole Mode Index (after the covariances with Niño3 and Niño4 at 0 month lead are removed), and the dashed-line box indicates the eastern pole of IOD. (b)-(e) Partial regression coefficients of SST anomalies averaged in different seasons onto September-November averaged Dipole Mode Index, only values exceeding the 95% significance level based on a two-tailed Student’s *t* test are shown. Units: °C /°C. Figures are plotted using MATLAB R2015b (<http://www.mathworks.com/>). The maps in this figure are generated by MATLAB R2015b with M_Map (a mapping package, <http://www.eos.ubc.ca/~rich/map.html>).


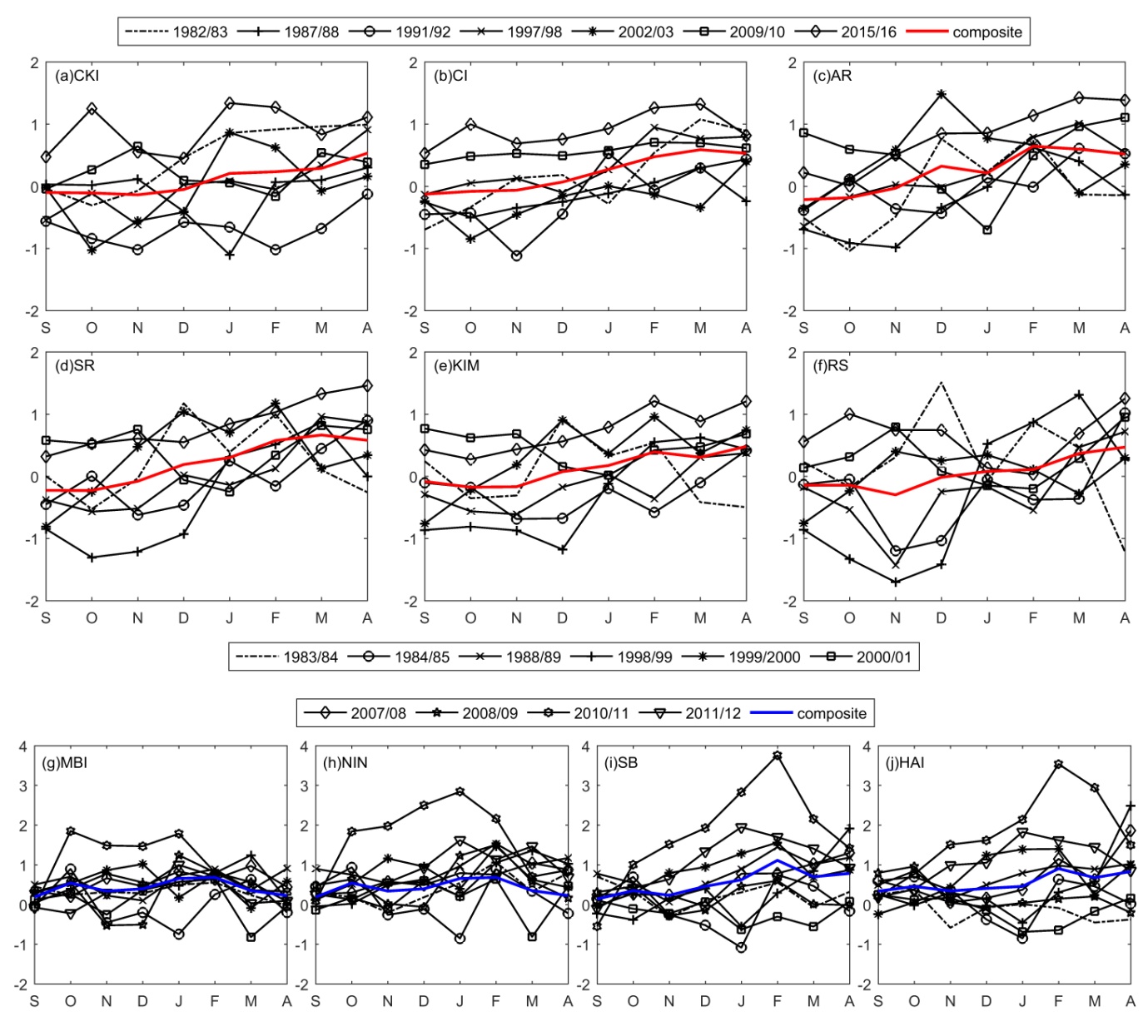


**Supplementary Fig. S3.** **(a)-(f)** SST anomaly series during each El Niño event at tropical reefs; **(g)-(j)** SST anomaly series during each La Niña event at subtropical reefs. Figures are plotted using MATLAB R2015b (<http://www.mathworks.com/>).


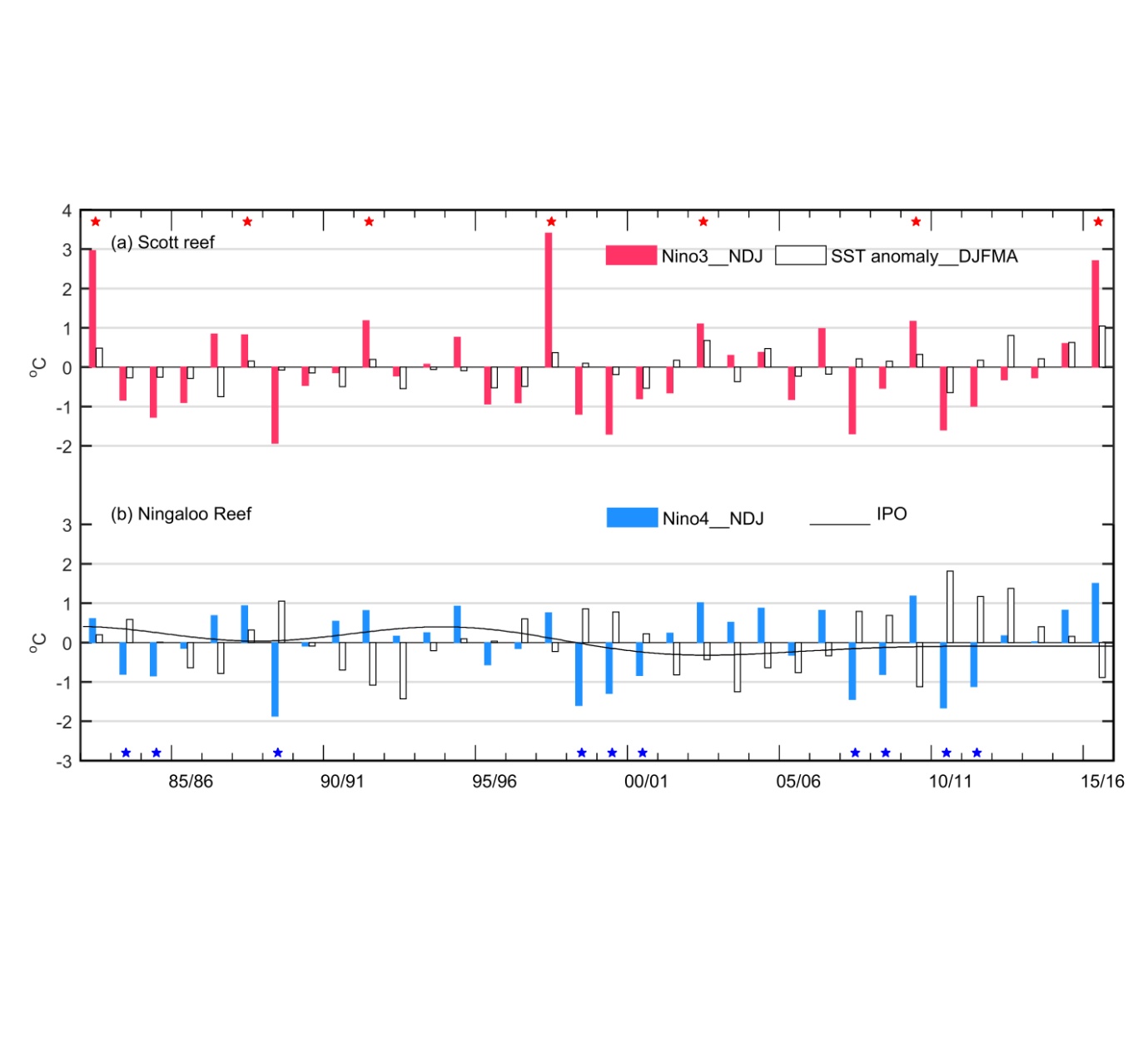


**Supplementary** **Fig. S4.** The same as Fig. 2k and 2l, only not standardised. Figures are plotted using MATLAB R2015b (<http://www.mathworks.com/>).


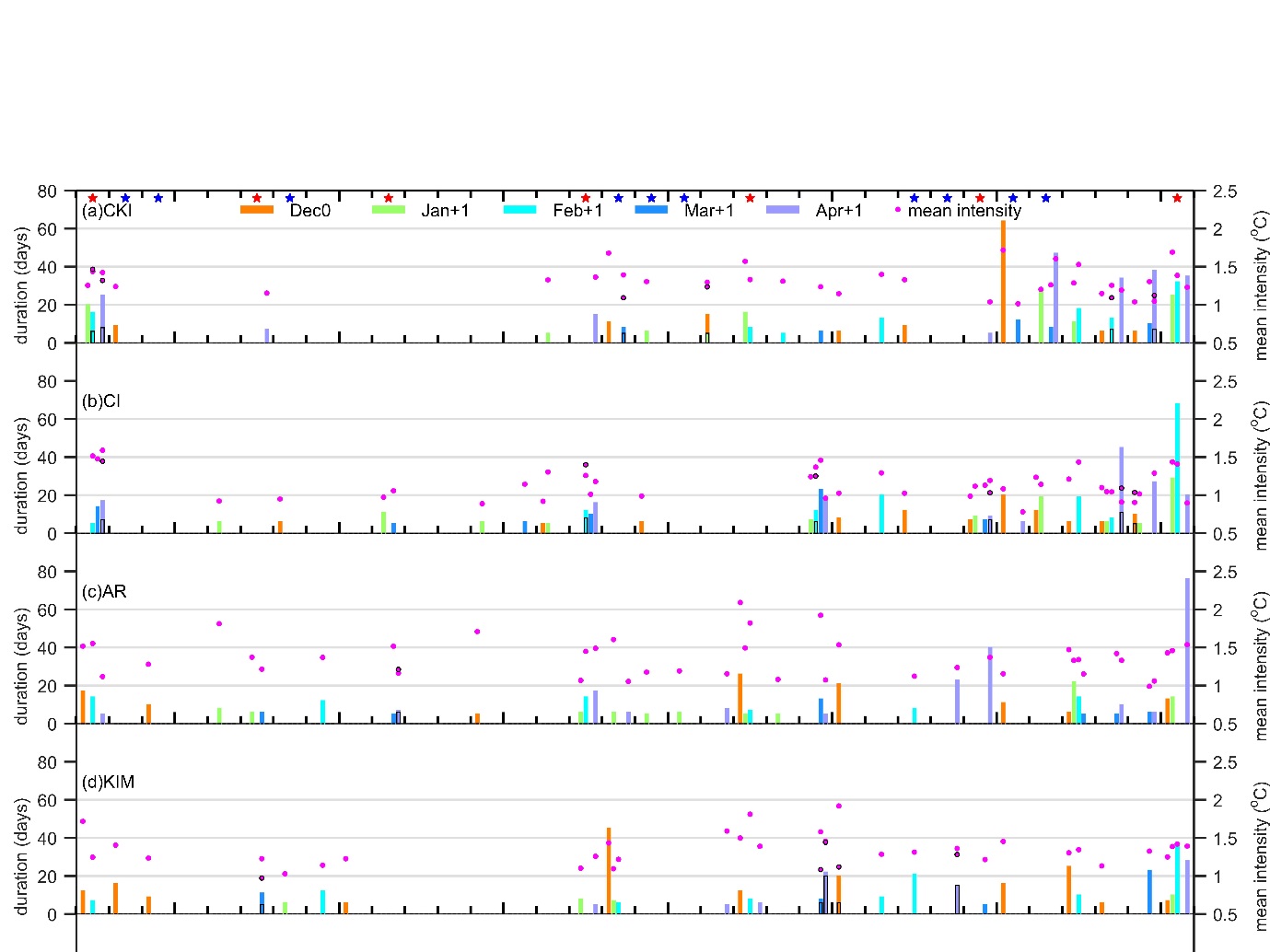

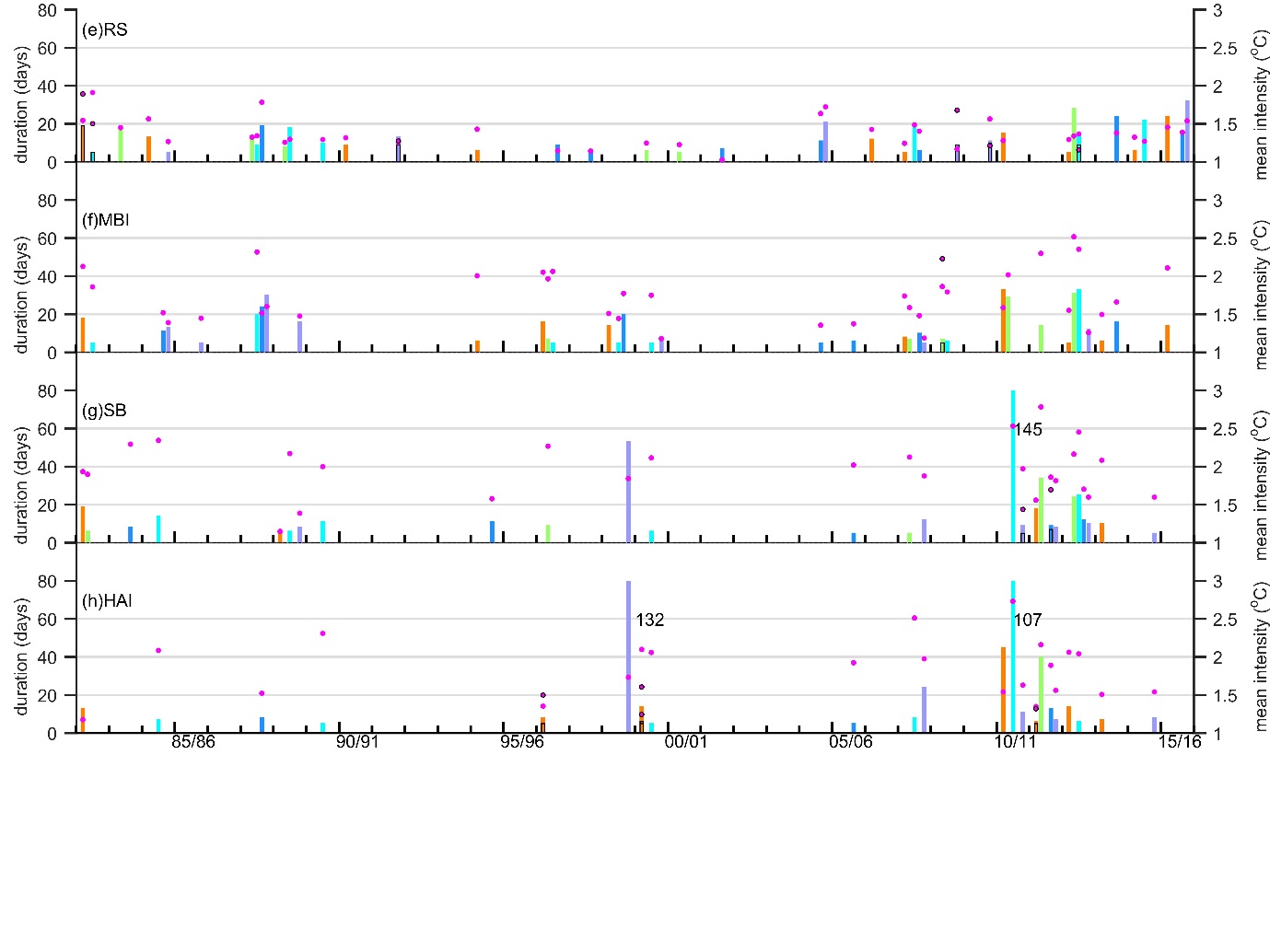
 **Supplementary Fig. S5.** Durations (bars) and mean intensities (dots) of the MHW events peaking in extended summer months (December to April) at each site. The time axis corresponds to the peak month of each MHW event. The red (blue) stars denote the developing years of El Niño (La Niña). Note the scales of mean intensity for **(a)-(d)** and **(e)-(h)** are different. Figures are plotted using MATLAB R2015b (<http://www.mathworks.com/>).


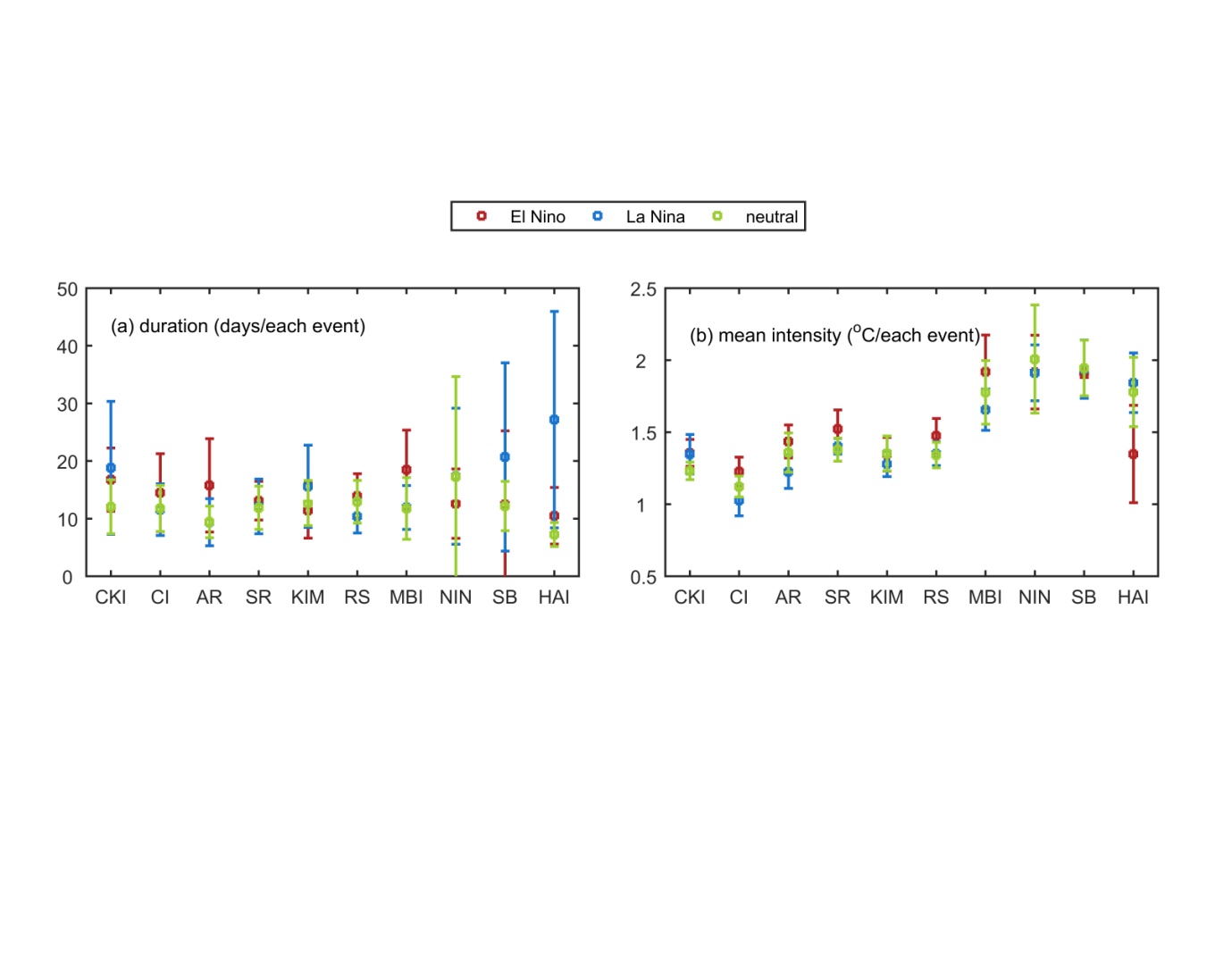


**Supplementary** **Fig. S6.** **(a)** Composited duration of each MHW event in the extended summer months (December to April) of El Niño and La Niña events. **(b)** Composited mean intensity of each MHW event in the extended summer months (December to April) of El Niño and La Niña events. The error bars denote 95% confidential intervals. Figures are plotted using MATLAB R2015b (<http://www.mathworks.com/>).


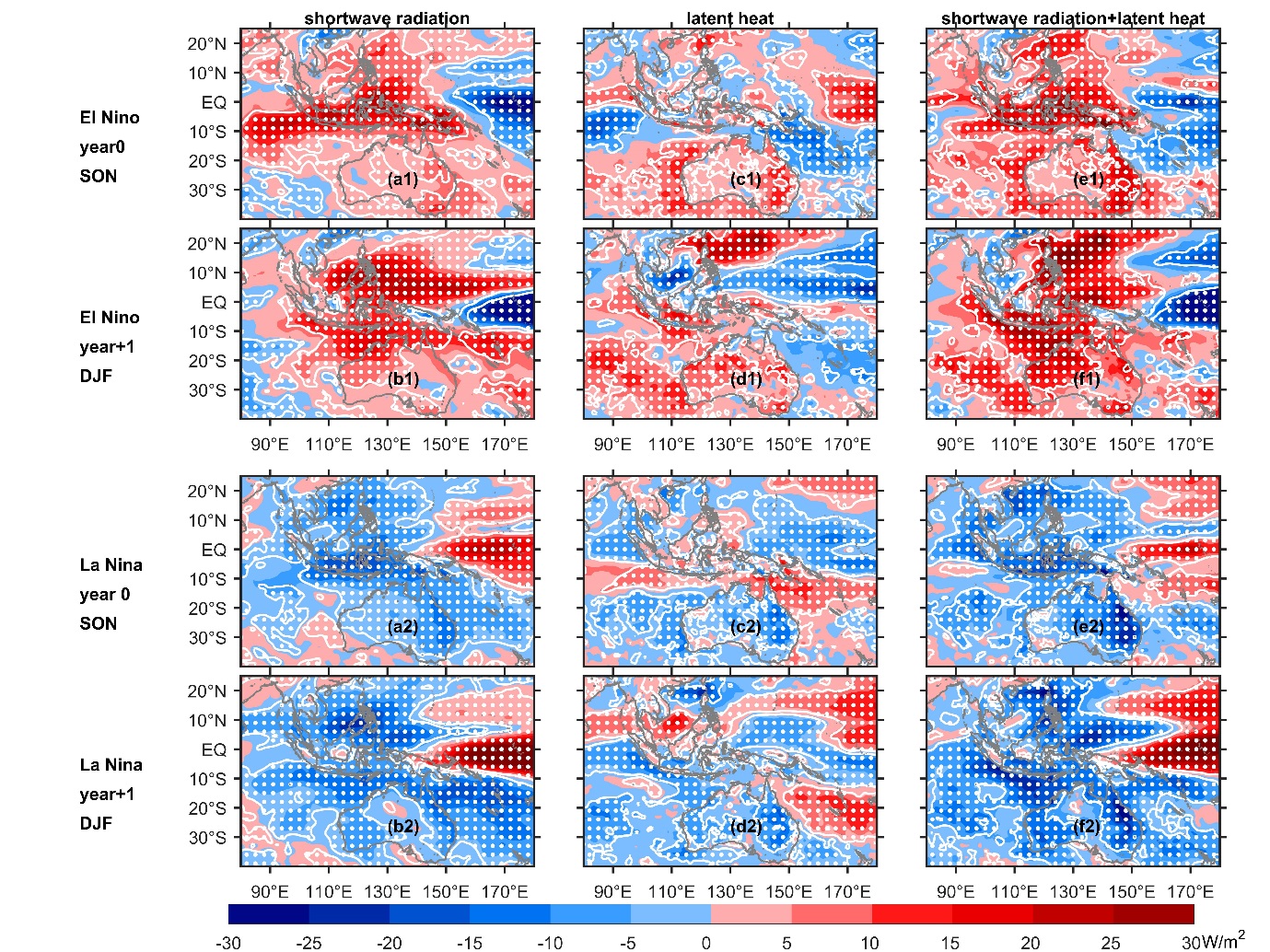


**Supplementary** **Fig. S7.** Composited patterns of shortwave radiation and latent heat fluxes during the developing and mature phases of El Niño and La Niña. The white contours and dots indicate anomalies exceeding the 90% significant level based on a two-tailed Student’s *t* test. Figures are plotted using MATLAB R2015b (<http://www.mathworks.com/>). The maps in this figure are generated by MATLAB R2015b with M_Map (a mapping package, <http://www.eos.ubc.ca/~rich/map.html>).


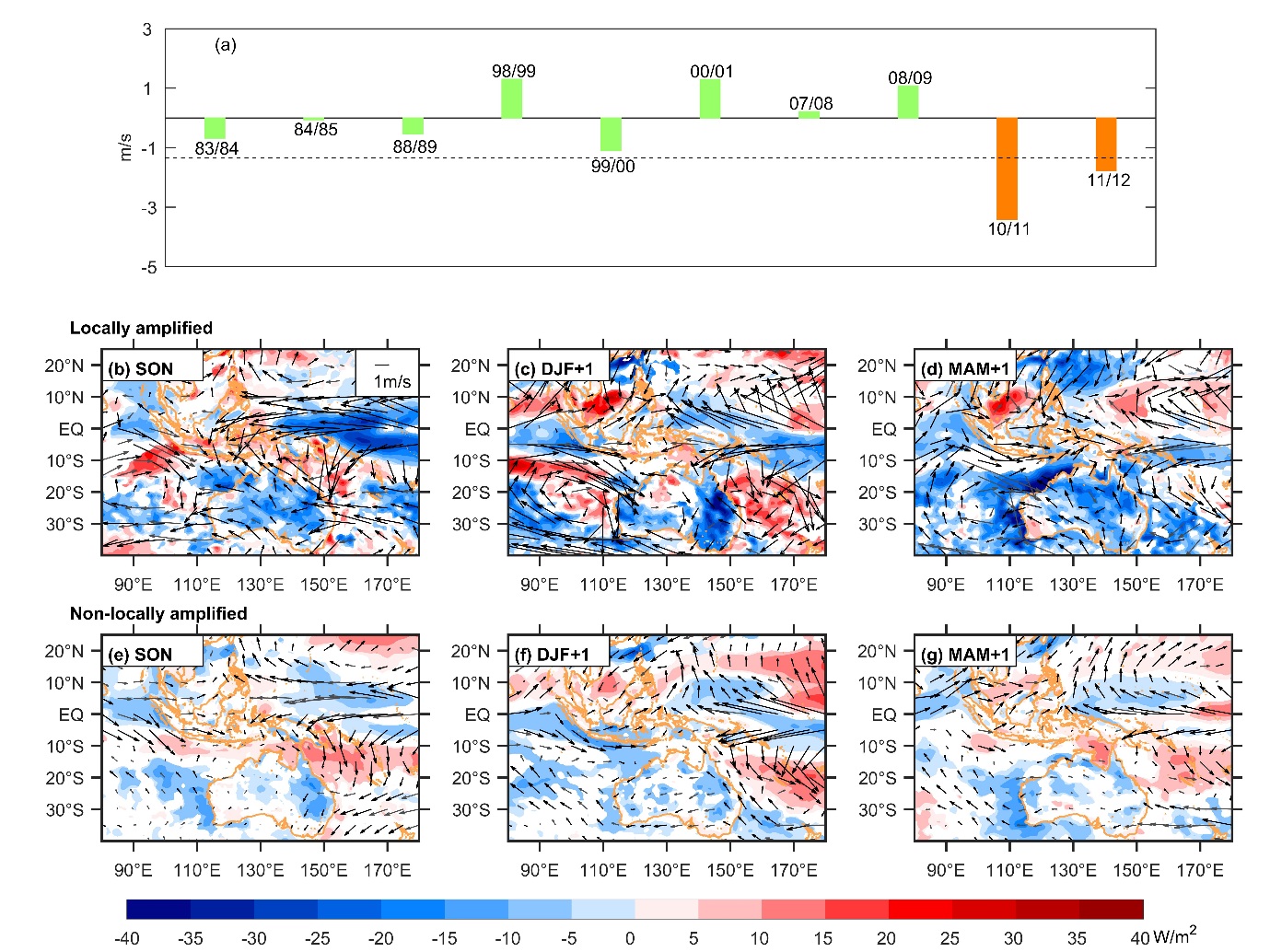


**Supplementary** **Fig. S8.** **(a)** December-February averaged coastal wind anomalies over the region 108–114°E, 28–22°S in the ten La Nina years. The orange and green bars denote locally and non-locally amplified years respectively. The dashed line is 0.9 sigma of the CWI of the La Niña years. **(b)- (d)** are composited latent heat flux and wind anomalies during locally amplified years. **(e)- (g)** are composited latent heat flux and wind anomalies during non-locally amplified years. Only anomalies exceeding the 90% significant level based on a two-tailed Student’s *t* test are displayed (black arrows for significant wind anomalies and grey for un-significant ones). Figures are plotted using MATLAB R2015b (<http://www.mathworks.com/>). The maps in this figure are generated by MATLAB R2015b with M_Map (a mapping package, <http://www.eos.ubc.ca/~rich/map.html>).
